# On the Treatment of Fevers

**Published:** 1802-01

**Authors:** 


### *To the Editors of the Medical and Phyſical Journal.*

GENTLEMEN,

IT is with concern I obſerve the continuance of a fever which has for ſome time paſt been more or leſs prevalent in this and neighbouring counties.

The

The flight manner in which the firft fymptoms occur has no doubt prevented the extermination of it; befides, what is ufually called a trifling cold, if neglected, will frequently run into or partake of fever, if it happens to be of the prevailing diforder.

I have not only fuffered twice from fever, but ample experience in practice enables me to recommend the following plan as the moft effectual, in averting the violence and preventing the progrefs of it.

Whenever there is reafon to fufpect an attack of fever, (which is often indifcriminately termed flow, nervous, or putrid, for it affumes a variety of ambiguous fymptoms at the commencement) give a bold dofe of calomel and James's powder, according to the age of the patient, and repeat the dofe as circumftances may require; wafh the whole body well with ftrong cold vinegar night and morning, then rub dry, and let the patient put on a flannel fhirt and drawers next the fkin; every time after the vinegar is ufed change the flannel dreffes, (never wafh them) but keep them expofed to the air through the day or night, and only warmed by the fire juft before being put on. If the bowels are particularly affected, keep cloths wet with cold vinegar conftantly applied.

Fumigate the room of the patient every night and morning with gunpowder moiftened, fo as to burn without explofion.

By this mode of treatment, if fever is not effectually deftroyed in the beginning, the fymptoms will be lefs violent and of fhorter duration; but when it has taken firm hold of the conftitution, it behoves the practitioner to be conftantly upon his guard, for although he may not prevent fever from going through its different ftages, he can very often *opportunely* check the moft alarming fymptoms.

I have been unable to prevail upon any patient to fubmit to ablution or immerfion in cold water, (as recommended by Dr. Currie of Liverpool) but I find no difficulty in advifing the ufe of vinegar, which is not only equally efficacious but more antifeptic than water, and there is lefs dread (upon the mind of the patient) about taking cold.

The limits of your Journal will not admit of my entering more fully upon this important fubject; I cannot, however, conclude without remarking, that the frequent renovation of the *human* as well as the *common* atmofphere *in a fick room*, is a fource of the greateft comfort to the patient and attendants, cuts off one material fource of communicating, and both are effectually produced by the plan now recommended.

*Southgate Street, Gloucefter,*

*Nov.* 13, 1801.

I remain, &c.

A MEDICAL PRACTITIONER.

